# Microarray Profiling and Co-Expression Network Analysis of Circulating lncRNAs and mRNAs Associated with Major Depressive Disorder

**DOI:** 10.1371/journal.pone.0093388

**Published:** 2014-03-27

**Authors:** Zhifen Liu, Xinrong Li, Ning Sun, Yong Xu, Yaqin Meng, Chunxia Yang, Yanfang Wang, Kerang Zhang

**Affiliations:** Department of Psychiatry, First Hospital of Shanxi Medical University, Taiyuan, People's Republic of China; University of Jaén, Spain

## Abstract

LncRNAs, which represent one of the most highly expressed classes of ncRNAs in the brain, are becoming increasingly interesting with regard to brain functions and disorders. However, changes in the expression of regulatory lncRNAs in Major Depressive Disorder (MDD) have not yet been reported. Using microarrays, we profiled the expression of 34834 lncRNAs and 39224 mRNAs in peripheral blood sampled from MDD patients as well as demographically-matched controls. Among these, we found that 2007 lncRNAs and 1667 mRNAs were differentially expressed, 17 of which were documented as depression-related gene in previous studies. Gene Ontology (GO) and pathway analyses indicated that the biological functions of differentially expressed mRNAs were related to fundamental metabolic processes and neurodevelopment diseases. To investigate the potential regulatory roles of the differentially expressed lncRNAs on the mRNAs, we also constructed co-expression networks composed of the lncRNAs and mRNAs, which shows significant correlated patterns of expression. In the MDD-derived network, there were a greater number of nodes and connections than that in the control-derived network. The lncRNAs located at chr10:874695-874794, chr10:75873456-75873642, and chr3:47048304-47048512 may be important factors regulating the expression of mRNAs as they have previously been reported associations with MDD. This study is the first to explore genome-wide lncRNA expression and co-expression with mRNA patterns in MDD using microarray technology. We identified circulating lncRNAs that are aberrantly expressed in MDD and the results suggest that lncRNAs may contribute to the molecular pathogenesis of MDD.

## Introduction

Major depressive disorder (MDD) is one of the most common psychiatric disorders and affects 10–15% of the general population with high levels of morbidity, disability, and mortality [1]. The heritability of MDD is approximately 40%, while the causal factors contributing to the other 60% are currently unknown [2], [3]. Epigenetic mechanisms mediate changes in gene expression that occur in response to diverse stimuli. Recent research has established that environmental events and behavioral experience induce epigenetic changes at particular gene loci and that these changes help shape neuronal plasticity and function and hence behavior contributed to the pathogenesis of depression [4].

Epigenetic mechanisms include DNA methylation, histone modification, chromosome remodeling and noncoding RNAs. Recently, the characterization of noncoding RNAs (ncRNAs) has become a fruitful area of research in MDD. Genome-wide surveys have revealed that eukaryotic genomes are extensively transcribed into thousands of long and small noncoding RNAs [5], [6]. Small ncRNAs are smaller than 200 bp in length and include the small interfering RNAs, microRNAs (miRNAs), and piwi-interacting RNAs [7], [8], [9]. Long ncRNAs (lncRNAs) are transcripts over 200 nucleotides in length that are found in both the cytoplasm and the nucleus [10]. LncRNAs, which represent one of the most highly expressed classes of ncRNAs in brain, are becoming increasingly interesting with regard to brain functions and disorders [11]. They regulate expression of neighboring protein-coding genes that have a pivotal role in development or disease progression, and also regulate gene expression via a trans-acting mechanism by associating with protein complexes, such as chromatin modifiers, transcription factors, splicing factors, or RNA decay machinery [12]. Therefore, analysis of the co-expression of lncRNAs and mRNA can help predict their functional role in the development of MDD as a foundation for further function and mechanism studies. Determining the targets of lncRNAs from their interactions with other biomolecules will be very beneficial to drug discovery [13] and clinic diagnosis as a biomarker. In particular, lncRNAs play a direct role in the regulation of genes involved in neural plasticity and cognitive function [14], [15], [16]. Indeed, an increasing number of studies have shown that lncRNAs are associated with several neurodegenerative and psychiatric disorders. For example, the lncRNA Disrupted in Schizophrenia 2 (DISC2) is antisense to and overlaps with the protein-coding transcript DISC1, which has been implicated in the pathogenesis of schizophrenia, bipolar depression, and autism spectrum disorder [17], [18], [19].

LncRNAs have broad spectrum functions in the normal brain development and function maintenance. Therefore, it is possible to determinate the underlying mechanisms in the development of MDD. Moreover, a recent study has demonstrated that noncoding RNAs are present in body fluids, such as serum, plasma, and urine, and can be readily measured using a variety of techniques, suggesting a great promise as a new class of psychiatry biomarkers due to their surprisingly high stability in plasma, association with disease states, and ease of sensitive measurement [20]. To investigate the potential role of lncRNA in regulating the development of MDD, we used a microarray analysis profiling to identify genome-wide lncRNAs expression and co-expression pattern with mRNAs in the peripheral blood of MDD patients. We further constructed gene-lncRNA co-expression networks associated with MDD to determine interaction patterns among genes with their related co-expressed lncRNAs.

## Materials and Methods

### Subjects and clinical assessments

This study recruited a total of ten patients and ten coupled matched controls. Summary data regarding the two groups are presented in Table 1. The patients were recruited from outpatients of the Department of Psychiatry at the First Hospital of Shanxi Medical University and the controls were recruited from the community nearby. All participants were Chinese of Han origin and came from the same geographical areas in northern China. All of the subjects met the following criteria: 1) aged between 30 and 50 years old; 2) presented with illness for more than 6 months; 3) had no previous history of neurological illnesses (such as Alzheimer's disease and Parkinson's disease) or other severe systemic diseases (such as heart disease and diabetes) and no participants was pregnant; 4) had no severe negative life event within 6 months before diagnosis; 5) had no unstable psychiatric features such as suicidal tendencies; 6) no history of head injury or Axis I psychiatric disorders except for the MDD diagnosis in the patient group; 7) diagnosis of MDD was established using the Chinese version of the Modified Structured Clinical Interview for DSM-IV, patient version (SCID-I/P) [21] according to interviews with patients and family members with at least two consultant psychiatrists. The inter-rater reliability kappa value of SCID was 0.81. The severity of major depressive disorder was assessed using the 17-item Hamilton Rating Scale (HAMD_17_) for depression; and 8) patients had no family history of Axis I psychiatric disorders except for the MDD diagnosis. All patients were on the first episode of MDD and had no history of antidepressants treatment with a minimum score of 21 on the HAMD_17_. The matched controls were interviewed using the Structured Clinical Interview for DSM-IV, nonpatient edition (SCID-I/NP) to ensure that they were excluded from diagnosis of MDD and other psychiatric diseases.

**Table 1:** Demographics and clinical characters for controls and patients with MDD.

Variable	MDD (n = 10)	Controls(n = 10)	*t* -value	*p*-value		
	Mean ± SD	Mean±SD				
Age	43.17±8.66	41.42±9.72	0.874	> 0.05		
Gender (M/F)	5/5	5/5				
Total HAMD_17_ score	25.37 (3.65)					
Illness duration (months)	16.4 (6.96)					

MDD: major depressive disorder. HAMD_17_: 17 item Hamilton Depression rating scale.

### Ethics Statement

The study was approved by the Ethical Committee for Medicine of the First Hospital of Shanxi Medical University, PR China. All participants and their legal guardians gave written informed consent.

### RNA isolation and purification

Peripheral blood (10 ml) was obtained just before breakfast. Mononuclear cells were removed within 1 h by density-gradient centrifugation and stored in Trizol at −80°C. Total RNA was isolated using Trizol according to the manufacturer's instruction. RNase-free DNase (Promega, Madison, WI, USA) treatments were conducted to remove genomic DNA contamination. The integration of RNA was confirmed by gel electrophoresis. RNA was further purified using an RNeasy mini kit (Qiagen, Valencia, CA, USA) according to the manufacturer's instructions.

### Expression microarray

The Glue Grant Human Transcriptome Array (GG-HTA, manufactured by Affymetrix Inc.) was used in this study. This array was developed for high-throughput clinical studies, allowing for comprehensive examination of gene expression and genome-wide identification of alternative splicing as well as detection of coding SNPs and noncoding transcripts [22]. The microarray can detect 34834 lncRNAs and 39224 transcript clusters (genes) curated from the most authoritative databases such as RefSeq and Ensembl as well as the literature. Repeat sequences and ncRNAs shorter than 200 bp were deleted. Each transcript was represented by 119 unique probes (on average) to improve statistical confidence. Each transcript was represented by a specific exon or splice junction probe that can identify individual transcripts accurately. The microarray analysis was performed by Gminix, Shanghai, PR China.

### Quantitative real-time PCR

The total RNA was extracted using TRIzol reagent (Invitrogen, USA) and then reversed transcribed using High Capacity RNA-to-cDNA Kit (Invitrogen, USA) according to the manufacturer's recommendations. The expression of up-regulated lncRNAs (FR344886, chr21:39641845-39641964, chr17:78355675-78355935, chr17:78354412-78354623) in all subjects included in this study was measured by real-time PCR using SYBR®Select Master Mix (Invitrogen, USA), and GAPDH was used as an internal control. For quantitative results, the expression of each lncRNA was represented as fold change using 2-ΔΔCt methods. The lncRNA expression differences between patients and controls were analyzed using Student's t-test. A value of *p*<0.05 was considered significant.

### Microarray analysis

Total RNA was used to synthesize double-stranded complementary DNA (cDNA) utilizing a random priming method, and then double-stranded cDNA was fragmented, labelled and hybridized to the Affymetrix GG-H Array. After hybridization and washing, processed slides were scanned with the Affymetrix GeneChip Scanner 3000 7G (Affymetrix, Santa Clara, CA). The Affymetrix Expression Console (version 1.2.1) implementation of RMA was used for quantile normalization and background correction. All gene level files were imported into Affymetrix Expression Console (version 1.2.1) and normalized by the quantile method. Combat Software was used to adjust the normalized intensity to remove batch effects. A random variance model (RVM) [23] *t*-test was applied to discriminate differentially expressed genes between controls and MDD patients because the RVM *t*-test can raise degrees of freedom effectively in the case of small samples. After false-discovery rate (FDR) analysis, we selected differentially expressed genes only if *p* value <0.05 and the change in expression was ≥1.5-fold. Hierarchical clustering was performed using EPCLUST. The microarray analysis was performed by Gminix, Shanghai, PR China.

### Functional group analysis

Gene Ontology (GO) analysis was applied to explore the functions of differentially expressed genes identified in this study. GO analysis organizes genes into hierarchical categories and can uncover gene regulatory networks on the basis of biological processes and molecular functions (http://www.geneontology.org) [24], [25]. Specifically, two-side Fisher's exact test was used to classify the GO category and the GO annotation list is greater than that expected by chance. A FDR [26] was calculated to correct the *p*-value. We computed *p*-values for GOs enriched among differentially expressed genes (the recommend *p*-value cut-off is 0.05). Pathway analysis was used to place differentially expressed genes according to Kyoto Encyclopedia of Genes and Genomes (KEGG), Biocarta and Reactome (http://www.genome.jp/kegg/). Fisher's exact tests were also used to identify pathways, and the threshold of significance was defined by the *p*-value. The enrichment was calculated in a similar manner as the GO analysis [27], [28].

### Co-expression network

We constructed gene co-expression networks to identify interactions among genes and lncRNAs [29]. Gene co-expression networks were built according to the normalized signal intensity of individual genes. The data were preprocessed by using the median gene expression value of all transcripts expressed from the same coding gene, without special treatment of the lncRNA expression value. We then screened the data for differentially expressed lncRNAs and mRNAs and removed these data from the dataset. For each pair of genes analyzed, we calculated the Pearson correlation and chose pairs (only lncRNA-mRNA) with significant correlations in order to construct the network [30]. To make a visual representation, only the strongest correlations (0.94 or greater) were included in these renderings. In this representation, each gene corresponded to a node and when two genes were connected by an edge it indicated a strong correlation (i.e., either positive or negative). The co-expression networks were drew using Cytoscape. Finally, the degree and normalized degree were calculated to examine the topological property of this graph. A degree was defined as the number of directly linked neighbors [31]. The normalized degree was calculated by dividing the max degree in the same network in order to reduce the difference between individuals.

The information regarding our data was submitted to the Gene Expression Omnibus, the accession number is GSE52790.

## Results

### Analysis of differentially expressed lncRNAs

**Figure 1:**
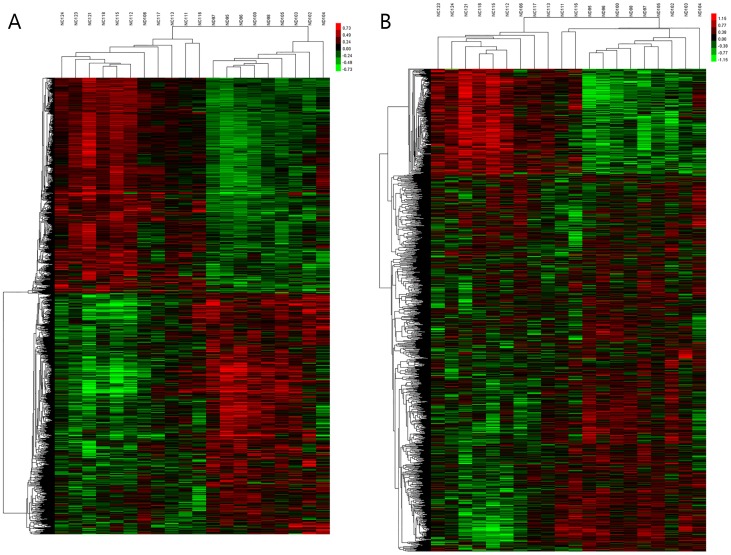
Profiles of differentially expressed genes in MDD compared to controls. (A) Differentially expressed lncRNAs and (B) differentially expressed mRNAs were subjected to hierarchical clustering. Red color indicates high relative expression and green color indicates low relative expression.

From the lncRNA expression profiles, differentially expressed lncRNAs were discriminated between MDD patients and controls. RVM *t*-test revealed 2007 differentially expressed lncRNAs between the two groups (Table S1), consisting of 1556 up-regulated lncRNAs and 441 down-regulated lncRNAs. Among these differentially expressed lncRNAs, 404 had higher fold change according to our filtering criteria (*p*<0.05, fold change ≥1.5), and lncRNAs in this subset were all up-regulated in the MDD group. A hierarchical clustering analysis was used to group lncRNAs based on their expression levels between samples. As shown in Figure 1, these lncRNAs displayed the general difference between MDD and controls. In our microarray datasets, lncRNAs were generally up-regulated in the MDD group, and in particular, FR344886 was the most significantly up-regulated lncRNA.

### Analysis of differentially expressed mRNAs

**Figure 2:**
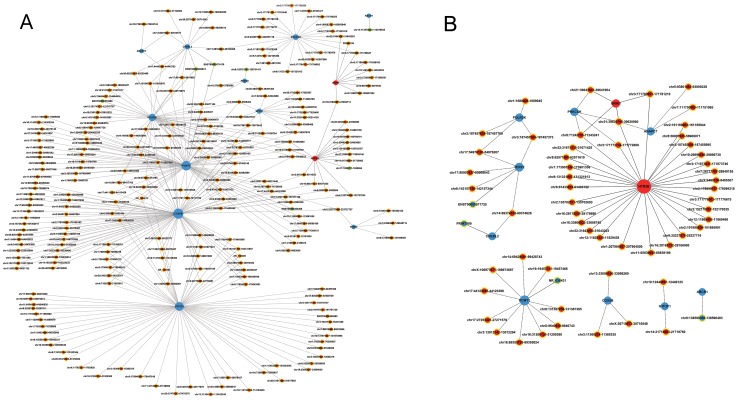
Co-expression sub-networks of MDD-associated genes and co-regulated lncRNAs. (A) Illustrates a sub-network derived from the MDD group and (B) illustrates a sub-network derived from the control group. Genes colored in red are up-regulated, genes colored in blue are down-regulated and genes denoted a yellow ring encode lncRNAs. Node size represents the node degrees.

From the analysis, 1766 differentially expressed mRNAs were found with comparisons between MDD patients and controls, which consisted of 759 up-regulated mRNAs and 1007 down-regulated mRNAs (Table S2). Of these, 157 mRNAs had higher fold change according to our filtering criteria (*p*<0.05, fold change ≥1.5) and all of these were up-regulated in the MDD group. Their distinct expression patterns are also presented in a hierarchical clustering analysis show in Figure 2.

### GO analysis and pathway analysis

A GO enrichment analysis of differentially expressed mRNAs was performed to identify GOs with higher confidence. Enrichment provides a measure of the significance of the function, and as the enrichment increases, the corresponding function is more specific, which helps us to identify GOs with more concrete functional description in the experiment [32]. To avoid missing the MDD-related genes, we performed these functional analyses for all of the differentially expressed mRNAs. We found that the most enriched GOs were associated with down-regulated transcripts, namely negative regulation of protein phosphatase type 2A activity (GO: 0034048) and notochord morphogenesis (GO: 0048570). In addition, the most enriched GO targeted by the up-regulated transcripts was “peptide secretion (GO: 0002790)” (Table S3 and S4). Importantly, this particular GO was associated with *ABCA1*, which is involved in antidepressant metabolism [33].

Pathway analysis indicated that two pathways were significantly enriched among the up-regulated transcripts and 68 pathways were significantly enriched among the under-regulated transcripts. The most enriched pathway was “metabolic pathways”, which was associated with 112 differentially expressed genes (Table S5). Several of these pathways were link to neurodevelopmental diseases, such as “Huntington's disease” (associated with 36 genes) and “Alzheimer's disease” (associated with 27 genes) (Table S6).

### Co-expression of lncRNAs and mRNAs

We first constructed general coding-noncoding gene co-expression networks in each of the two separate groups (i.e., MDD patients and controls) with all differentially expressed lncRNAs and mRNAs from this study. Those lncRNAs and mRNAs that had Pearson correlation coefficients equal to or greater than 0.94 were selected, and from that a network was constructed in each of the above mentioned groups using Cytoscape program. The overall structures of constructed co-expression networks of MDD patients and controls were significantly different (Fig S1, S2) in the number of nodes and connections. Specifically, the co-expression network for MDD was composed of 499 nodes and 1050 connections between 125 lncRNAs and 374 coding genes, while the network for the controls was composed of 304 nodes and 415 connections between 111 lncRNAs and 193 coding genes. The network of MDD included 802 negative pairs and 248 positive pairs, while the network derived from controls included 346 negative pairs and 70 positive pairs (Table S7). Within the network analysis, parameter of a degree is the simplest and most important measure of the centrality of a gene within a given network, which represents the relative importance of this gene. For each node, the degree and normalized degree are presented in each network in Table S7, which shows the weight of each node in the whole network. The top 15 lncRNAs with the largest degree changes between MDD and control are shown in Table 2.

**Table 2:** The top 15 lncRNAs with largest degree differences in MDD co-expression network compared with controls.

lncRNA	dir	ND- Degree	ND_K	NC_ Degree	NC_K	DiffK (ND-NC)	|DiffK|
**chr21:39630795-39630900**	up	0	0	4	0.1333	−0.1333	0.1333
**chr8:71242705-71243241**	up	0	0	3	0.1	−0.1	0.1
**chr14:69374533-69374626**	up	0	0	2	0.0667	−0.0667	0.0667
**chr17:54976106-54976207**	up	0	0	2	0.0667	−0.0667	0.0667
**chr21:39641845-39641964**	up	0	0	2	0.0667	−0.0667	0.0667
**chr3:171772744-171772869**	up	0	0	2	0.0667	−0.0667	0.0667
**chr3:187457269-187457373**	up	0	0	2	0.0667	−0.0667	0.0667
**chr3:171780951-171781210**	up	1	0.0115	2	0.0667	−0.0552	0.0552
**chr10:75873456-75873642**	up	4	0.0460	0	0	0.0460	0.0460
**chr10:874695-874794**	up	4	0.0460	0	0	0.0460	0.0460
**chr3:47048304-47048512**	up	4	0.0460	0	0	0.0460	0.0460
**chr1:29506417-29506566**	up	3	0.0345	0	0	0.0345	0.0345
**chr1:92783513-92783593**	up	3	0.0345	0	0	0.0345	0.0345
**chr15:92482784-92482899**	up	3	0.0345	0	0	0.0345	0.0345
**chr16:28178775-28178879**	up	3	0.0345	0	0	0.0345	0.0345

ND: MDD group; NC: Control group; dir: direction of differential regulation in MDD compared to control; k: normalized degree; DiffK: difference between MDD and controls; |DiffK|: absolute value of DiffK.

To identify the lncRNAs network specifically involved in the molecular pathogenesis of MDD, we subsequently used a regulatory subset of differentially expressed mRNAs (totally 17, listed in Table 3), which were detected in this study and have been previously shown to be associated with the etiology and pathology of MDD from several depression-related studies [3], [34], [35], [36], [37], [38], [39], [40], [41], [42], [43], [44], [45]. These sub-networks of co-expression were constructed and shown in Table S8. Similar to the general networks described above, the nodes and connections in this MDD sub-network were significantly more abundant than that in the controls sub-network (Fig 2). The top 15 lncRNAs with the largest degree changes between MDD and control are shown in Table 4. Three MDD up-regulated lncRNAs, namely chr10:874695-874794, chr10:75873456-75873642, and chr3:47048304-47048512 were connected to four differentially regulated genes in the MDD sub-network but these connections were lost in the control's sub-network. These identified lncRNAs may play a vital regulatory role in molecular regulation of MDD.

**Table 3:** Genes with previous associations to MDD represented among the differentially expressed genes identified in this study.

Symbol	Name	Synonyms	Locus
*ABCB1*	ATP-binding cassette, sub-family B (MDR/TAP), member 1	*CD243, GP170, ABC20*	7q21.12
*CCND2*	cyclin D2	n/a	12p13
*CD3E*	CD3e molecule, epsilon (CD3-TCR complex)	n/a	11q23
*FBXO8*	F-box protein 8	*FBX8, FBS*	4q34.1
*GRIK1*	glutamate receptor, ionotropic, kainate 1	n/a	21q22
*HTR2B*	5-hydroxytryptamine (serotonin) receptor 2B	*5-HT(2B), 5-HT2B*	2q36.3-q37.1
*OLIG1*	oligodendrocyte transcription factor 1	*BHLHB6, bHLHe21*	21q22.11
*PRKCH*	protein kinase C, eta	*PKC-L, PKCL*	14q23.1
*COX5B*	Cytochrome c oxidase subunit 5B	*COXVB*	2q11.2
*GTF2F1*	general transcription factor IIF	*BTF4,RAP74, TF2F1, TFIIF*	19p13.3
*CREBL2*	cAMP responsive element binding protein-like 2	n/a	12p13
*PCMT1*	protein-L-isoaspartate (D-aspartate) O-methyltransferase	*PIMT*	6q24-q25
*PRKCSH*	protein kinase C substrate 80K-H	*AGE-R2, G19P1, PCLD, PKCSH, PLD1*	19p13.2
*SPCS1*	signal peptidase complex subunit 1 homolog (S. cerevisiae)	*HSPC033, SPC1, SPC12, YJR010C-A*	3p21.1
*TERF2*	telomeric repeat binding factor 2	*TRBF2, TRF2*	16q22.1
*POLR2K*	polymerase (RNA) II (DNA directed) polypeptide K, 7.0kDa	*ABC10-alpha,RPABC4, RPB10alpha,RPB12, RPB7.0,hRPB7.0, hsRPB10a*	8q22.2
*ANAPC7*	anaphase promoting complex subunit 7	*APC7*	12q24.11

MDD-associated genes are derived from [3], [34], [35], [36], [37], [38], [39], [40], [41], [42], [43], [44], [45]

**Table 4:** The top 15 lncRNAs with largest degree differences in MDD sub-network compared with controls.

lncRNA	dir	ND- Degree	ND-K	NC- Degree	NC-K	DiffK (ND-NC)	|DiffK|
**chr21:39630795-39630900**	up	0	0	4	0.1333	−0.1333	0.1333
**chr8:71242705-71243241**	up	0	0	3	0.1	−0.1	0.1
**chr14:69374533-69374626**	up	0	0	2	0.0667	−0.0667	0.0667
**chr17:54976106-54976207**	up	0	0	2	0.0667	−0.0667	0.0667
**chr21:39641845-39641964**	up	0	0	2	0.0667	−0.0667	0.0667
**chr3:171772744-171772869**	up	0	0	2	0.0667	−0.0667	0.0667
**chr3:187457269-187457373**	up	0	0	2	0.0667	−0.0667	0.0667
**chr3:171780951-171781210**	up	1	0.0115	2	0.0667	−0.0552	0.0552
**chr10:75873456-75873642**	up	4	0.0460	0	0	0.0460	0.0460
**chr10:874695-874794**	up	4	0.0460	0	0	0.0460	0.0460
**chr3:47048304-47048512**	up	4	0.0460	0	0	0.0460	0.0460
**chr1:29506417-29506566**	up	3	0.0345	0	0	0.0345	0.0345
**chr1:92783513-92783593**	up	3	0.0345	0	0	0.0345	0.0345
**chr15:92482784-92482899**	up	3	0.0345	0	0	0.0345	0.0345
**chr16:28178775-28178879**	up	3	0.0345	0	0	0.0345	0.0345

ND: MDD group; NC: Control group; dir: direction of differential regulation in MDD compared to control; k: normalized degree; DiffK: difference between MDD and controls; |DiffK|: absolute value of DiffK.

### Real-time Quantitative PCR Validation

**Figure 3:**
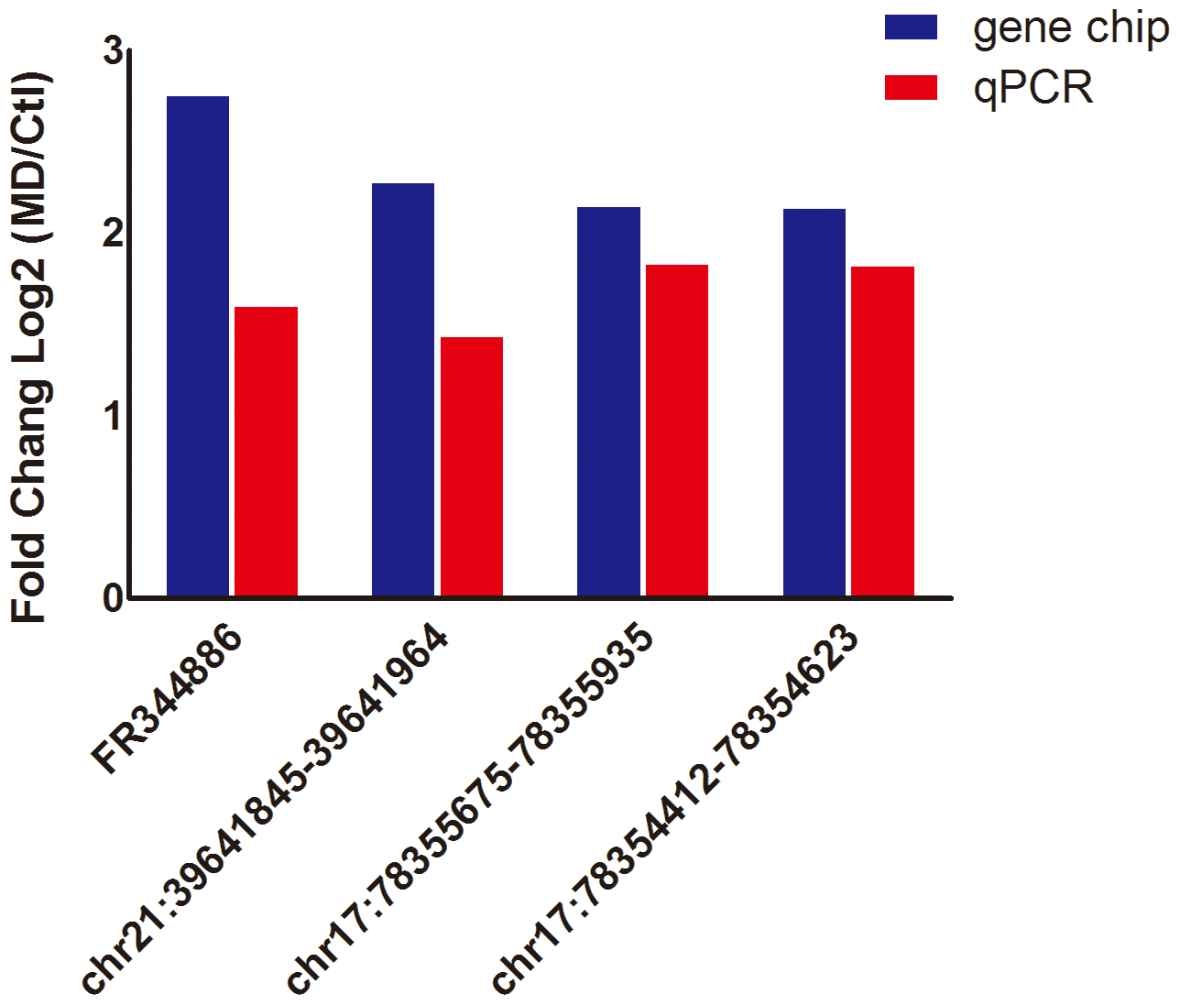
Comparison between microarray and quantitative real-time PCR results. Four differentially expressed lncRNAs were validated by qPCR. The heights of the columns in the chart represent the log-transformed median fold changes (MD/Control) in expression across ten samples (p<0.05).

We examined the expression of these four lncRNAs (up-regulated: FR344886, chr21:39641845-39641964, chr17:78355675-78355935, chr17:78354412-78354623) in two sets using qPCR. The primers are listed in Table S9. These data supported a strong consistency between the qPCR result and microarray data (Figure 3).

## Discussion

This study for the first time examined the expression of lncRNAs in MDD patients using microarray analysis and found that lncRNAs that were differentially expressed in peripheral blood of MDD patients compared to the controls. From these microarray expression profiles, we found that 2007 lncRNAs were differentially expressed, which consisted of 1556 up-regulated and 441 down-regulated lncRNAs, and of these, 404 up-regulated lncRNAs had a higher fold change. Meanwhile, 1766 differentially expressed mRNAs were identified consisting of 759 up-regulated and 1007 down-regulated mRNAs, and of these, 157 were up-regulated with a higher fold change. Among these differentially expressed mRNAs, 17 genes were documented as depression-related gene in previous studies (Table 3). Therefore, our mRNA expression data are quite consistent with earlier studies. Further analysis of the co-expression network of lncRNAs and mRNAs indicated significantly different patterns in the MDD group compared to the control group, whereby both nodes and connections in the MDD sub-network were significantly more abundant than that in the controls sub-network. These results suggest lncRNAs involvement, which may contribute to the molecular pathogenesis of MDD through regulating gene expression. To the best of our knowledge, only a few mRNA expression microarray studies associated with MDD have been reported using peripheral blood at a genome-wide level to date [46], [47]. Those studies identified changes in a variety of genes, such as decreased transcriptional engagement in the cell proliferation, DNA replication, and repair processes in women postpartum depression, or increase expressed of Type I interferon signalling genes in recurrent major depression. Some other studies have analyzed candidate genes expression using qPCR methods, the results of which seem inconsistent or are difficult to replicate [48]. The reasons may be due to a variety of factors such as the experimental approach, target tissue, and disease state in different studies. In our co-expression study, although mRNAs were largely down-regulated, but it seems that up-regulated genes commonly had a larger change. One possible explanation for this result is that the expression of these genes may be low, which limits the range of the inhibition effect. Therefore, further studies will be needed to resolve these inconsistencies.

Furthermore, we used GO and pathway analyses to identify biological functions enriched among the differentially expressed coding-genes. We found that these genes are mainly involved in basic metabolic processes and signal transduction. The pathway analysis identified two pathways associated with the up-regulated transcripts and 68 pathways associated with the down-regulated transcripts, which suggests that the pathology of MDD is mainly associated to an inhibitory process of expression and regulation of multiple genes. Interestingly, these pathways are associated with basic metabolic processes that are enriched in neurodevelopment disease and associated with Huntington's disease, Alzheimer's disease, Parkinson's disease and alcoholism. Moreover, neurodevelopment plays a important role in the etiology of depression [49] and previous studies have shown that these CNS related diseases and depression partially share similar behavioral, neurotransmitter, and genetics changes. [50], [51], [52], [53], [54].

Although the detailed mechanisms of how these genes are involved in MDD remain largely unknown, the differentially expressed lncRNAs may contribute to MDD by regulating these coding-genes. Our co-expression network analysis indicated a more complex regulatory relationship between lncRNAs and mRNAs in MDD compared to the controls. LncRNAs chr10:874695-874794, chr10:75873456-75873642, and chr3:47048304-47048512 in the MDD sub-network were each connected to four differentially expressed mRNAs. This result suggests that these lncRNAs may play an important role in their corresponding networks or signalling, and therefore may contribute to the molecular regulation of MDD. The dysregulation of noncoding RNAs and/or an altered noncoding RNA response has been suggested to play a critical role in the etiology and pathophysiology of MDD through the processes of synaptic plasticity, neurogenesis, and stress responses [55]. LncRNAs participate in the regulation of gene expression by targeting transcription factors, initiating chromatin remodelling, directing methylation complexes, and blocking nearby transcription [56]. Moreover, lncRNAs have been shown to be involved in neural plasticity, cognitive function and the development of some neuropsychiatric disorders [57]. Several recent reports have demonstrated that lncRNA expression is altered in psychiatric disorders. For example, the down-regulation of Gomafu lncRNA leads to alternative spicing patterns in schizophrenia [58]. Our current understanding of lncRNA regulation in brain function and disease is in its infancy in the future, several approaches can be employed to determine their biological functions, including lncRNA silencing and structure disruption [59]. This study provides new insight into the mechanisms of psychiatric disorders such as MDD.

In this study we also observed that the majority of MDD-related differentially expressed genes were present in our network with a higher normalized degree, which validates the use of gene co-expression networks as an effective means to identify the role and interaction of related transcripts in gene co-expression network.

In order to reduce the interference of other psychiatric disease and factors with the results, we used rigorous recruitment criteria for the patient selections. We excluded individuals in the depressive phase of bipolar disorder, those experiencing geriatric depression and depressive states caused by severe adverse life events. The individuals included in this study were therefore selected to reduce confounding variable and were representative of MDD.

Besides, our study used peripheral blood as the source of biological material for microarray analysis. Although important differences in gene expression certainly exist between biological fluids and brain tissues, peripheral blood cells share more than 80% of the transcriptome with brain, colon, heart, kidney, liver, lung, prostate, spleen, and stomach [60]. However, the expression levels of many classes of biological processes have been shown to be comparable between whole blood and prefrontal cortex [61]. Our study suggests that the judicious use of gene expression analysis in peripheral blood may be a useful surrogate for studying gene expression in the CNS when it has been determined that the relevant gene is expressed in both regions [62]. A genome-wide analysis of lncRNA stability found that only a minority of lncRNAs are unstable [20]. The detection of circulating lncRNAs in peripheral blood not only represents a new layer of complexity in the molecular architecture of MDD, but it also reveals the potential to use them as diagnostic markers and therapeutic targets. Although the emerging application of circulating lncRNAs for disease diagnosis is restrained by our limited knowledge of the stability of these lncRNAs and the specific regulatory networks in which they participate, the use of individual lncRNAs in clinical cancer research has already begun to be tested [63].

In conclusion, we report here for the first time that circulating lncRNAs are differentially expressed in MDD patients compared to demographically-matched controls. Three lncRNAs were detected as potential regulatory factors in MDD, most likely through interactions with coding transcripts. Further work is needed to understand the biological functions and molecular mechanisms of specific lncRNAs implicated in MDD.

## Supporting Information

Figure S1 **Co-expression network of differentially expressed mRNAs and lncRNAs in the MDD group (S1) and the controls (S2).** Genes colored in red are up-regulated, genes colored in blue are down-regulated and genes shaped the quadrate encode lncRNAs, the round denoted mRNAs. Node size represents the node degrees.
(PNG)

Figure S2 **Co-expression network of differentially expressed mRNAs and lncRNAs in the MDD group (S1) and the controls (S2).** Genes colored in red are up-regulated, genes colored in blue are down-regulated and genes shaped the quadrate encode lncRNAs, the round denoted mRNAs. Node size represents the node degrees.
(PNG)

Table S1 **Profile of differentially expressed lncRNAs.**
(XLS)

Table S2 **Profile of differentially expressed mRNAs.**
(XLS)

Table S3 **Significantly different GOs targeted by differentially expressed genes.**
(XLS)

Table S4 **GO-Enrichment histogram.**
(XLS)

Table S5 **Significantly different pathway targeted by differentially expressed genes.**
(XLS)

Table S6 **Up and down-regulated pathway—(-LgP) histogram.**
(XLS)

Table S7 **The co-expression network between lncRNAs and mRNAs in MDD.**
(XLSX)

Table S8 **Co-expression sub networks of MDD-associated genes and co-regulated lncRNAs.**
(XLSX)

Table S9 **The primers of four lncRNAs for the quantitative real-time PCR. experiment.**
(DOC)
