# Supplementary material for: Microarray Profiling and Co-Expression Network Analysis of Circulating lncRNAs and mRNAs Associated with Major Depressive Disorder
Source: PLoS One. 2014 Mar 27;9(3):e93388. doi: 10.1371/journal.pone.0093388 (PMC3968145; doi:10.1371/journal.pone.0093388)
Supplement: Table S9 — The primers of four lncRNAs for the quantitative real-time PCR. experiment. (DOC) [file pone.0093388.s011.doc]

>FR344886|AP004318|U4 spliceosomal RNA|U4_snRNA|RF00015 Rfam v8.1|Homo sapiens|140nt

AGCTTTGCCCAGTGGCAACATCATAACCAGTGAGGTTTATACAGGGTGCAATTATTGCTAACTGAAAACT

TTTCCTAATACCCACCATGATGACTTGAAATATAGTCGGCATTGGCAATTTTGGATAGTCTCTATGGAGA

LNC1-P1: 5' TAACCAGTGAGGTTTATACAGGGTG 3'

LNC1-P2: 5' ACTATCCAAAATTGCCAATGCC 3'

>hg19_dna range=chr21:39641845-39641964 5'pad=0 3'pad=0 strand=- repeatMasking=none

GCTGCAAAGGCAATGGCAGTGAAGTGGGGAAGCATACAGGAGCAATTGAA

CAGGGCGGGGGGCCTTGCTGGGGAGCGGTGGGAAGAAGTGTGGGTGAAGC

AGAGTCAGGCCATAGGGTTC

LNC2-P1: 5' CAAAGGCAATGGCAGTGAAGT 3'

LNC2-P2: 5' TGACTCTGCTTCACCCACACTTC 3'

>hg19_dna range=chr17:78355675-78355935 5'pad=0 3'pad=0 strand=- repeatMasking=none

TGATTGATCCTTTGACACGCACGCTCTGTGTGGACGACACGTGCTCCCAG

CATGGTAGGGGAATGACCTACGGGGCTGACTTCGGAACTGAAGACTCCCC

ATATATGCACACTGAACACTTGCGTGCAGACGCTCCCGAATACCGCAGGT

CTTAGCCAGGCAGGGGTGAATGGAGGGAACAGAGCAGCTCCTTCCAGCCT

CTGGGCAAGCAGAGTGCTTCCTTTTTCCAGCCCCAGGCATCGCCTTCCCT

AGACACGGTTT

LNC3-P1: 5' GAATGACCTACGGGGCTGACT 3'

LNC3-P2: 5' CTGCTCTGTTCCCTCCATTCA 3'

>hg19_dna range=chr17:78354412-78354623 5'pad=0 3'pad=0 strand=- repeatMasking=none

GAACGAGAGAACACAAGACAGAGGCTGAGGCTGGCCGCTTTGGCACGCTG

CACTTTCTCAGCACTTGGAGTGAGGCTTACGGCTGAGAAGAGAGTTATGT

GAAGAGTACTTGCTGTTCCACAAAGCCTGGACCATGTTCCTAAGAGTCTC

TCTCATGATAGACTCGCCTTTCCGGTGGACCAGACCCGACCGCATTCTCA

GCGTCAGGTGGC

LNC4-P1: 5' TTGGAGTGAGGCTTACGGCT 3'

LNC4-P2: 5' CACCGGAAAGGCGAGTCTA 3'
